# An ongoing case-control study to evaluate the NHS breast screening programme

**DOI:** 10.1186/1471-2407-13-596

**Published:** 2013-12-13

**Authors:** Nathalie J Massat, Peter D Sasieni, Dharmishta Parmar, Stephen W Duffy

**Affiliations:** 1 Centre for Cancer Prevention, Wolfson Institute of Preventive Medicine - Room 009, Queen Mary University of London, London EC1M 6BQ, UK

**Keywords:** Breast cancer, Case–control, Incidence, Mortality, Overdiagnosis, Advanced stage, Bias

## Abstract

**Background:**

In England, a national breast screening programme (NHSBSP) has been in place since 1988, and assessment of its impact on breast cancer incidence and mortality is essential to ensure that the programme is indeed doing more good than harm. This article describes large observation studies designed to estimate the effects of the current programme in terms of the benefits on breast cancer incidence and mortality and detrimental effect in terms of overdiagnosis. The case-control design of the cervical screening programme evaluation was highly effective in informing policy on screening intervals and age ranges. We propose innovative selection of cases and controls and gathering of additional variables to address new outcomes of interest and develop new methodologies to control for potential sources of bias.

**Methods/Design:**

Traditional case-control evaluation of breast screening uses women who have died from breast cancer as cases, and women known to be alive at the time of case death as controls. Breast screening histories prior to the cases’ date of first diagnosis are compared. If breast screening is preventing mortality from breast cancer, cases will be characterised by a lesser screening history than controls. All deaths and incident cases of primary breast cancer in England within each 2-year study period will be included in this ongoing evaluation. Cases will be age- and area-matched to controls and variables related to cancer treatment and breast tumour pathology will be obtained to investigate the interplay between screening and treatment, and the effect of screening on incidence of advanced stage disease. Screening attendance at other national screening programmes will also be collected to derive superior adjustment for self-selection bias.

The study is registered and has received full ethics approval.

## Background

Breast cancer is the most commonly diagnosed cancer among women in the UK, accounting for a third of all female cancer cases, and the second most common cause of cancer death: in England in 2010, the age-standardised rate per 100,000 women-years was 125.7 for incidence and 24.3 for mortality; in addition, in situ breast cancer, that is Ductal Carcinoma In Situ (DCIS) and Lobular Carcinoma In Situ (LCIS), was diagnosed at a rate of 18 per 100,000 women in 2010 (CRUK, Accessed 28 March 2013 [1]).

In the early nineties, meta-analyses of the randomised controlled trials (RCTs) confirmed the efficacy of mammographic screening for reducing primary breast cancer mortality, and led to the implementation of breast screening programmes in several regions of Europe. One of the most mature and comprehensive of those is the English National Breast Screening Programme (NHSBSP) which has been in place in England since 1988. A major issue to be addressed by the Department of Health’s (England) Policy Research Unit in Cancer Awareness, Screening and Early Diagnosis (PRU), is to evaluate the policy of mammography screening as delivered in the current NHSBSP in terms of benefits on mortality from and on incidence of invasive primary breast cancer, and harms from the most adverse outcome of breast screening. Overdiagnosis is the detection of a breast cancer that would never have been clinically identified in the lifetime of the woman if she had not been screened. In the post-RCT epoch, analytical observational studies are the design of choice.

A key feature of case–control evaluation should be that the study is nested within a well defined cohort of individuals, with screening exposure data prospectively recorded. This confers a reliability and interpretability comparable with a prospective evaluation. The basic premise of the case–control audit for a screening programme is as follows: if screening ‘works’ in preventing the relevant clinical outcome, then those who experience the outcome (cases) will be characterised by a lesser history of screening than those who do not (controls). Cases and controls are compared with respect to screening history, and the reduction in odds of the relevant clinical outcome is estimated for various measures of exposure to screening by using the ratio of the odds (Verbeek and Broeders, 2010 [2]). The traditional case–control evaluation of breast screening takes women aged 50-70/75 years at diagnosis as cases, who have died from primary breast cancer, and women known to be alive at the time of death of the cases as controls. Controls are usually matched approximately for date of birth and, possibly, geographical area. Screening histories prior to the date of first primary breast cancer diagnosis of the cases are then compared between cases and controls. Previous case–control evaluations of the NHSBSP have been undertaken in the East Anglia and West Midlands areas. They found that screening in the NHSBSP was associated with a reduction in breast cancer deaths, at least as great as that obtained in the randomised controlled trials of mammographic screening. In the East Anglia case–control study, the odds ratio for risk of death from primary breast cancer in women who attended at least one routine screen (i.e. potentially including the screen leading to diagnosis) compared to those who did not attend was 0.52 (95% CI: 0.32, 0.84; Allgood et al., 2008 [3]). In the West Midlands case–control study, the corresponding odds ratio was 0.43 (95% CI: 0.27, 0.67; Allgood, Warwick & Duffy et al., unpublished data). However, an earlier case–control study in Wales (Fielder et al., 2004 [4]) found a more modest effect (odds ratio = 0.75, 95% CI 0.49–1.14). The design of the East Anglia and West Midlands studies were very similar in that they identified cases among women diagnosed since 1995 and 1994, respectively, to avoid choosing cases resulting from the accumulation at the prevalent round of the screening programme, and to minimize exposure (screening) opportunity bias (Walter, 2003 [5]). This, however, may have introduced a bias towards the inclusion of cases with short survival (women had to have died before the end of 2004 and 2005, respectively), and consequently overestimated the benefit of screening. In contrast, the Wales study identified cases among women diagnosed between 1991 and 2000, and was therefore dominated by prevalence screen data. The odds ratios of those studies were all adjusted for self-selection bias using the relative rate of breast cancer deaths among non-attenders in the screening group versus those in the control group, estimated from the RCTs (Duffy et al., 2002 [6]). This adjustment is at best approximate as the RCTs of screening took place many years ago, in countries other than the UK, and any decision to participate in screening was made in the absence of evidence regarding the benefits and harms of screening.

Data from demographic and cohort population studies were recently used to provide estimates of overdiagnosis in the service screening setting: in England and Wales, overdiagnosis was estimated to be between 3.3 and 10% of the expected cumulative incidence from age 50 to 79 in the absence of screening after correction for self-selection bias and lead-time bias (Paci et al., 2012 [7]). Evaluation on a large scale is needed to confirm those bias corrections.

Mortality from breast cancer has been declining in the UK population since the early 1990s (CRUK, Accessed 28 March 2013 [1]). This fact coincides with the introduction of both the national screening programme, and new adjuvant therapies, and the respective role of those two factors in mortality reduction is a matter of debate (Cancer Intervention Surveillance Modelling Network (CISNET) Breast Cancer Collaborators, 2006 [8]).

In view of the above, there is a need for a large case–control study to (1) evaluate the effect of the NHSBSP on primary breast cancer mortality, with improvements to design and analysis methodology for coping with bias, notably self-selection bias: we propose to do this by developing a procedure using data from different cancer screening programmes. In addition, there is a need to (2) quantify the relative contributions of screening attendance, tumour attributes and treatment variables on case fatality in primary breast cancer, (3) assess the effect of screening attendance on primary breast cancer incidence, (4) estimate the rate of overdiagnosis due to screening, and (5) estimate the effect of screening attendance on incidence of advanced stage primary breast cancer. This final check may firstly give a result earlier than mortality and therefore confer greater statistical power, and secondly be used as a check on the mortality result. Inconsistency between the observed effect on mortality and that on advanced stage disease will indicate that further work is required to deal with residual bias.

Meeting the above aims will entail retrieving more information than in the traditional case–control evaluation of screening. It will include live incident cases as well as dead ones, and diseased controls as well as disease-free ones. Additional variables such as cancer treatment, breast tumour pathology, and screening attendance at other national screening programmes will also be collected.

This will be an ongoing biennial evaluation to ensure that the programme continues to deliver the anticipated health benefit, and to potentially improve the programme by identifying good and bad practices.

## Methods/Design

### Study design & objectives

This study is comprised of two sets of retrospective matched case–control studies: the first set involves dead cases of primary breast cancer (cases A), and the second set, recently diagnosed (or incident) cases of primary breast cancer, dead or alive (Cases C). The dead cases will be matched with two different sets of controls: two general population controls who were alive at the case’s date of death (Controls B), and one diseased control who was alive at the case’s date of death and had had invasive primary breast cancer (screen-detected, interval and never screened will all be included) at the case’s date of first diagnosis (Controls E). The incident cases will be matched to two controls from the general population (Controls D). Subsets of cases and matched controls will be selected for the purpose of each case–control evaluation objective (see *Participants*: *Selection of cases & controls* for details).

***Case–control study 1 (CC1)*** will be a matched comparison of the breast cancer dead cases (A) with the general population controls (B) with respect to screening exposure strictly prior to the case’s date of first diagnosis (set as pseudo-diagnosis date in controls). There will be two controls per case. This design should allow the evaluation of screening exposure modalities in relation to mortality from breast cancer.

***Case–control study 2 (CC2)*** will be a matched comparison of the breast cancer dead cases (A) with the diseased controls (E) with respect to screening exposure strictly prior to the case’s date of first diagnosis (set as pseudo-diagnosis date in controls). There will be one control per case. This design should enable assessment of the interplay between screening attendance, tumour attributes and treatment variables on fatality from breast cancer.

***Case–control study 3a (CC3a - Incidence)*** will be a matched comparison of recently diagnosed/incident **invasive** breast cancer cases (C) with the general population controls (D) free of invasive disease with respect to screening exposure strictly prior to the case’s date of index diagnosis (set as pseudo-diagnosis date in controls). There will be one or two controls per case. This design should allow the evaluation of screening attendance in relation to breast cancer incidence.

***Case–control study 3b (CC3b - Overdiagnosis)*** will be a matched comparison of recently diagnosed/incident invasive and in situ breast cancer cases (C) with the general population controls (D) **free of invasive** and of **invasive or in situ** disease, respectively, with respect to screening exposure strictly prior to the case’s date of **index** diagnosis (set as pseudo-diagnosis date in controls), and will be stratified by age at diagnosis/pseudo-diagnosis. There will be one or two controls per case. This design should allow the evaluation of overdiagnosis due to screening attendance.

***Case–control study 4 (CC4)*** will be a matched comparison of recently diagnosed/incident **advanced stage** breast cancer cases (C) with the general population controls (D) free of advanced stage disease with respect to screening exposure strictly prior to the case’s date of **index** diagnosis (set as pseudo-diagnosis date in controls). There will be one or two controls per case. This design should allow the evaluation of screening attendance on incidence of advanced stage breast cancer. In principle, one should include all advanced breast cancers as cases, i.e. whether advanced at diagnosis or whether subsequently progressed to advanced; however, in practice it will not be possible to identify those that subsequently progress. This analysis will be compared to the analysis of fatal breast cancers to see whether advanced breast cancer can be used as a surrogate endpoint when studying the impact of breast screening on breast cancer mortality.

**Figure 1:**
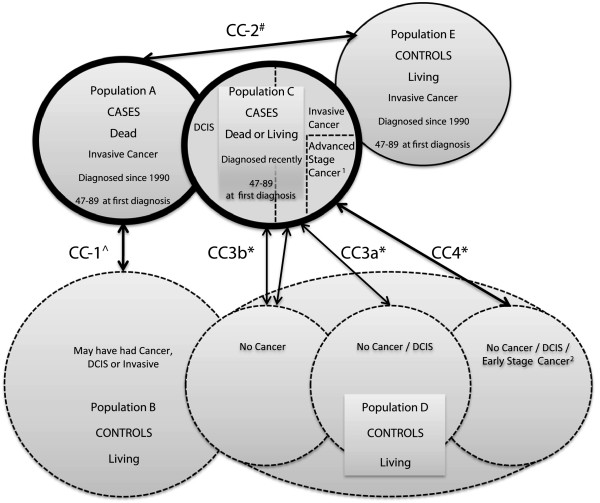
**Overview of the case–control study designs.** Legend: Thick circles, cases; Thin circles, controls; Full border, individuals with cancers; Dotted border, general population individuals, most of whom have not had cancer, but may have had DCIS or Early Stage Cancer^2^). ^ Match on date of birth within 1 month either side; 2 controls per case. * Match on date of birth within 1 month either side; 1 control per case. # Match on date of birth within 1 month either side, date of first diagnosis within 2 months prior to *and including* the case’s date of first diagnosis; 1 control per case. ^1^Advanced stage is defined as lymph node positive or tumour size >20 mm or both. ^2^Early stage is defined as lymph node negative, and tumour size ≤20 mm.

An overview of the proposed case–control studies and corresponding populations are presented in Figure 1.

### Setting & source population

The background, implementation and organisation of the NHSBSP are described in detail elsewhere (Advisory Committee on Breast Cancer Screening, 2006 [9]). For this study, all women who were invited to participate in the breast screening programme in England (from 1988 onwards), and who did not express dissent to their records being used for evaluation purposes, will be targeted.

The evaluation will begin with a pilot phase in collaboration with the Knowledge & Intelligence team London, KIT London (formerly Thames Cancer Registry), using cases that died from or were diagnosed with breast cancer between 1st of January 2008 and 31 of December 2009 in London. The main phase of the study will then be undertaken, which will cover the whole of England or, in the case of CC2, those regions (i.e. previous cancer registries) for which tumour attributes and treatment variables will be available from 1st of January 2010 - in collaboration with the Office for National Statistics (ONS). Each main phase evaluation will cover a 2-year period.

### Participants: selection of cases & controls

#### ***CC1 & CC2***

*Population of dead cases (A)* All women who died of primary breast cancer (as stated in part 1 of the death certificate) age 47–89, within the specified 2-year study period will be selected. Cases of women with only in situ primary breast cancer records (i.e. no invasive record) will be included, but Death Certificate Only (DCO) cases will be excluded. Case A women will have had their date of first diagnosis of primary breast cancer (in situ or invasive) age 47–89 and since 1990, and for subsequent phases of this evaluation (main phase onwards), they will not have been included as cases in previous evaluation periods, although they may have been included as controls, and may also be selected as a case C in the same evaluation.

*Population of cases A-matched general population controls (B)* For each case A, two women will be selected among the general population in the Health & Social Care Information Centre (HSCIC) database at Exeter who, are registered in the same National Health Applications and Infrastructure Services (NHAIS) system as the case at the case’s date of first diagnosis, are alive at the case’s date of death, and were born within 1 month either side of the case’s date of birth. Control B women may have had primary breast cancer (in situ or invasive) at the case’s date of first diagnosis. For subsequent phases of this evaluation (main phase onwards), they will not have been included as population controls B, E, or D in previous evaluation periods, although they may be included as a controls E or D within the same evaluation period.

*Population of cases A-matched diseased controls (E)* For each case A, one woman will be selected among the CR population who was alive at the case’s date of death, and was born within 1 month either side of the case’s date of birth. DCO cases will be excluded. Control E women will have had primary invasive breast cancer diagnosed within 2 months prior to *and including* the case’s date of first diagnosis and: this is necessary to ensure that the control survived for at least as long as the cases. She will have been registered in the same Cancer Network (CN) at the case’s date of first diagnosis. If no control E is found using this criterion, the date range will be expanded to 3 months prior to the case’s date of first diagnosis (or 6 months prior, if the case’s age at first diagnosis is less than 50 or over 70). For subsequent phases of this evaluation (main phase onwards), they may have been included as population controls B or D in previous or within the same evaluation periods, and may be included as a case C within the same evaluation period.

#### ***CC3a, CC3b & CC4***

*Population of incident cases (C)* All women who have had primary breast cancer (in situ or invasive), diagnosed age 47–89 within the specified 2-year study period will be selected. The **index diagnosis** will be either a first or a subsequent tumour and, therefore, women may have had a previous history of in situ or invasive primary breast cancer (i.e. prior to the date of their index diagnosis). Case C women may have died from primary breast cancer (as stated in part 1 of death certificate) age 47–89 during the specified 2-year study period, or afterwards. Cases of women with in situ primary breast cancer records only (i.e. no invasive record) will be included, but DCO cases will be excluded. For subsequent phases of this evaluation (main phase onwards), they will not have been included as cases in previous evaluation periods, although they may have been included as controls, and may also be selected as a case A in the same evaluation.

*Population of cases C-matched general population controls (D)* For each case C, two women will be selected from the general population in the HSCIC database at Exeter who, are registered in the same NHAIS system as the case at the case’s date of index diagnosis, are alive at case’s date of index diagnosis, and were born within 1 month either side of the case’s date of birth. For subsequent phases of this evaluation (main phase onwards), they will not have been included as population controls B, E, or D in previous evaluation periods, although they may be included as controls B or E within the same evaluation period.

Incident cases (C) and matched general population controls (D) will be selected *a posteriori* by the PRU, using tumour information, to suit the purpose of each case-study (see Figure 1).

**For CC3a**, incident cases (C) will be restricted to those women with invasive breast cancer, and matched general population controls (D) will be restricted to those women who have never had invasive primary breast cancer, but may have had in situ primary breast cancer prior to *and including* the case’s date of index diagnosis.

**For CC3b**, all incident cases (C) will be selected. For in situ cases, matched general population controls (D) will be restricted to those women who never had primary breast cancer (in situ or invasive) prior to *and including* the case’s date of index diagnosis; for invasive cases, to those women who never had invasive primary breast cancer, but may have had in situ primary breast cancer.

**For CC4**, incident cases (C) will be restricted to those women with advanced stage (defined below) primary breast cancer, and matched general population controls (D) will be restricted to those women who have never had advanced stage primary breast cancer, but may have had in situ or early stage primary breast cancer prior to *and including* the case’s date of index diagnosis. Breast cancer will be defined as “advanced stage” if tumour size is larger than 20 mm, or if at least one regional lymph node is affected, or if both, and as “early stage” if tumour size is equal to or smaller than 20 mm and no regional lymph node is affected. For the purpose of data collection, we have used an inclusive definition of “advanced stage”, but we will also analyse the data by Stage to assess the association between screening and metastatic breast cancer for instance, and using only those cases which are node positive, as this is generally accepted as an important prognostic factor and as a predictor of the effect of screening on mortality (Smith et al., 2004 [10]).

If no matched control is found for a particular case (A or C) with a date of birth within 1 month either side of the case’s date of birth, the range will be expanded to 3 months either side (or 6 months either side if the case’s age at index diagnosis of primary breast cancer is under 50 or over 70).

All cases and controls will appear on the HSCIC database at Exeter. We will aim to select female cases and controls who have been registered with the NHS by age 47 (determined by either date of first registration on local NHAIS system or prior cervical/bowel screening history), and have had available data on both breast and cervical (for women aged 47–64), and, possibly bowel (for women aged 60–69) screening strictly prior to their date of first diagnosis/pseudo-diagnosis (that is, they have been invited to screening at least once), although this may not be practicably feasible for dead cases and matching controls with older age at death. Patient breast, cervical, and bowel (for the main phase) screening history, primary breast tumours attributes and treatment data will be retrieved for all cases and controls, as well as deprivation quintile.

A detailed description of the algorithm to be used for the selection of cases and controls is available from the authors.

### Data sources & collection

Dead cases (A) and matching diseased controls (E) will be extracted by KIT London, and matching general population controls will be retrieved by the HSCIS at Exeter. Recently diagnosed cases will be extracted by KIT London, and matching general population controls will be retrieved by the HSCIS at Exeter. Tumour pathology variables will be obtained from the National Cancer Data Repository (NCDR) via KIT London, but may only be available for certain regions (i.e. previous cancer registries). Breast screening data will be obtained, as for cervical and bowel screening variables, from the HSCIS. Bowel screening variables will not be sought for the pilot phase.

The data will be checked and cleaned by the PRU Senior Data Manager, transferred to separate Oracle tables, and stored on a UNIX server kept in a secure server room within the Wolfson Institute of Preventive Medicine. Access to the Oracle database is from PCs on the secure QMCR network using SQLNET.

All data will be processed in accordance with NHS Information Governance guidelines.

### Variables

A list of all the variables to be retrieved during the ongoing evaluation study is presented in Table 1.

**Table 1:** Variables of interest

**Patient characteristics**	**Primary breast tumour attributes**	**Screening data**
	**(all primary breast tumours)**	
Patient unique ID	Tumour unique ID	NHS registration by age 47
NHS number	Tumour status (in situ or invasive)	Screening office/centre
Gender	ICD10 code	Episode date
Case or Control	Date of tumour diagnosis	Screen / Test date
Date of birth	Tumour detection mode	Screen / Test outcome
Patient status (alive or dead at end of study period)	Treatment	
Cause of death – Breast cancer (where applicable)	Tumour size	
Date of death (where applicable)	Number of lymph nodes	
Age at death (where applicable)	Stage	
Date of tumour diagnosis / pseudo-diagnosis	Grade	
Age at tumour diagnosis / pseudo-diagnosis		
Cancer registry		
Geographical area		
IMD (Postcode-based)		

Information retrieved regarding living area on which to match cases and controls will vary depending on the study (pilot or main phase) and time of evaluation. NHAIS information will be obtained from HSCIC for Health for all cases and controls used for CC-1, CC-3a/b and CC-4 studies, while CN information will be used by KIT London to match cases and controls for CC-2.

### Study size / Power calculation

The main phase of the study will cover the whole of England with biennial accrual of potentially thousands of cases (circa 18,000 (CRUK, Accessed 28 March 2013 [1])), for which statistical power will not be an issue for any of the case studies, and for which there will be considerable precision. The large sample size will also allow subset evaluation, notably by time since last screen. The sample size for the pilot study was estimated to enable sufficient power to answer the primary objectives addressed by CC1 and CC2 with relative confidence. Results of the pilot with respect to CC3a, CC3b and CC4 objectives will provide the prior estimates currently lacking to perform power calculations.

**CC1.** For the matched comparison of the breast cancer dead cases (A) with the general population controls (B), assuming an odds ratio of 0.7 for the event of ever attending screening (Broeders et al., 2012 [11]), around 900 dead cases and 1800 living controls would confer 90% power to detect such an effect size at the 5% significance level when using a 2-sided test (Machin et al., 1997 [12]).

**CC2.** For the matched comparison of the breast cancer dead cases (A) with the diseased controls (E), no estimate of the odds ratio is available, but it can be assumed to be 0.70 or less for the event of ever attending screening, to be at least consistent with CC1. With only one control per case, 1200 / 900 dead cases and the same number of controls will be required to obtain 90% and 80% power, respectively.

Missing data for some of the breast tumour attributes and treatment can be close to 40-50%, and the pilot sample size will be inflated accordingly. Sensitivity analyses will be conducted for various possible scenarios of non-random missing status.

We are therefore planning to collect pilot data from at least 1400 dead cases (A), together with 2800 general population controls (B) and 1400 diseased controls (E) matched for these. We will also collect at least 1400 recently diagnosed cases (C) and 2800 general population matched controls (D). There will be some degree of overlap between populations (A, C), (C, E) and (B, D).

### Bias & Effect modification

The case–control design is potentially prone to a number of biases (Walter, 2003 [5]), in particular some that could confer a bias *in favour of screening*, and which are addressed either at the design stage by choosing appropriate selection criteria, or at the analysis stage by using suitable statistical methods.

#### ***Ascertainment bias***

Among the screen-detected cases who die with primary breast cancer as a contributing cause on their death certificate (including DCO cases), there are those who would have died without any diagnosis of breast cancer, had they not been screened, forming part of overdiagnosis due to screening (Duffy, 2010 [13]). This introduces a bias *against screening* when assessing the impact of screening attendance on mortality, although unlikely to be a large one. It will be addressed at the analysis stage by a series of sensitivity analyses, assuming a range of plausible magnitudes of the bias. This bias may also qualify interpretation of the results from an analysis assessing the impact of time since last screen on the case–control status among screen-detected cancer patients.

In contrast, women with multiple co-morbidities who may be less likely to have breast cancer recorded as cause of death may be less likely to be screened. Consequent exclusion of those women from our datasets would confer a bias *against screening*.

#### ***Exposure (screening) opportunity bias***

Once diagnosed with breast cancer, the cases come under clinical management and do not continue with routine breast screening as before. The controls, however, may continue to attend screening up to the time of the case being entered into the study. This potential bias is addressed at the design stage by giving the controls a pseudo-diagnosis date that is the same as that of their matched case and screening history is only considered up to that date (Connor et al., 2000 [14]). This is in fact an over-correction and results in a bias *against screening*. The fact of the case having necessarily a diagnosis of breast cancer and a control usually not having such a diagnosis, induces an artificially higher retrospective probability of screening exposure in the cases, a bias *against screening* (i.e. the screen-detected cases will always have this screen recorded at diagnosis, whereas the large majority of controls will not at pseudo-diagnosis). Simply excluding the detection screens from the histories would bias the results *in favour of screening*. When assessing the effect of ever having been screened, the major driver of this bias is prevalence screening, and for a mature programme with approximately 6 incidence screens for each prevalence screen, this bias is likely to be small. However, this bias may remain when using other measure of screening exposure. The extent of screening opportunity bias will be investigated by applying an analytical correction to the odds ratio using the method by Duffy et al., 2008a [15], and by performing sensitivity analyses in which the date of pseudo-diagnosis for controls will be extended by up to 4 years, the estimated average sojourn time for each screen-detected case, to counteract the artificially higher retrospective probability of screening exposure in cases (Connor et al., 2000 [14]).

But the major sources of bias in case–control studies are potential self-selection and lead-time biases (Puliti et al., 2011 [16]). Length bias will also be discussed. Note that lead-time and length biases will only affect analyses assessing the effect of screening on survival among cases (i.e. case-study 2), but not those assessing its impact on population outcomes (incidence or mortality).

#### ***Self-selection bias (or non-compliance bias)***

Women who accept the invitation to screening (attenders) may have an a priori better health status (and a lower underlying risk of dying from breast cancer) compared with women who do not (non-attenders), and therefore are less likely to die from the disease. We would anticipate that this will confer a bias *in favour of* screening. Although this bias in favour of screening is unavoidable at the design stage, it can be approximately corrected for in the statistical analysis. In women diagnosed with breast cancer aged 47–52 (and their controls) we will look at two factors: “(invited and) screened”, “invited but not screened” compared to “not invited”. The odds ratio in “invited but not screened” can be used to adjust for the self-selection bias using the method by Duffy et al. (2002) [6]. This method cannot be used in older women as they will all have been invited.

The method developed by Duffy et al. (2002) [6] will also be used to correct the estimated odds ratio using data on participation in the NHSCSP (cervical) and/or NHSBCSP (bowel) screening programmes, rather than data on participation from the RCTs of mammographic screening. In the absence of self-selection, the relative risk of primary breast cancer (death/incidence) associated with breast screening for non-attenders at cervical or bowel screening would be expected to be equal to the relative risk in cervical or bowel screening attenders (after adjusting for breast cancer screening). In practice, the relative risk of primary breast cancer has been shown to be about 10% lower in non-attenders to RCTs (Duffy et al., 2002 [6]). The crucial element in correcting for self-selection bias is the risk ratio (termed “Dr”) for non-attenders versus attenders to cervical or bowel screening. This will be calculated for women who attended breast screening at least once and for women who did not attend a single screen, to control for attendance at breast screening. A range of denominator values for “Dr” will be assessed for sensitivity. One could compare primary breast cancer rates (i) in women who have had bowel/cervical screening - but not breast screening -with women who have had neither bowel/cervical nor breast screening; and (ii) in women who have had *both* bowel/cervical and breast screening with women who have only had breast screening. Data for women with age at diagnosis/ pseudo-diagnosis between 47 and 64 year-old who would have been invited to both the breast and cervical screening programmes will be used. Alternatively, data for women with age at diagnosis/pseudo-diagnosis between 60 and 69 year-old who would have been invited to both the breast and bowel screening programmes will be used. Also, in women under age 50, one could compare primary breast cancer rates in women who have, or have not had, cervical screening, hence by-passing the positive confounding between uptake of the various screening programmes.

Breast screening coverage of women aged 53–70 in England was 77% at 31st March 2012 (The HSCIC Breast Screening Programme – England report, 2011-2012 [17]).

In addition, self-selection will be addressed at an individual woman level by adjusting the regression model for participation in the other screening programmes, with careful adjustment for the confounding between attendances in different programmes: participation will be recorded as “Currently screened”, “Formerly screened” and “Never screened”, enabling all women to be included in the analysis.

For the pilot phase, a single self-selection factor will be estimated as the data covers one Strategic Health Authority (SHA) only (London); for the national phase, regional factors may be estimated to assess variation in self-selection between regions. This approach will be compared with adjusting the regression model for potential confounders, such as history of cervical/bowel screening, and socioeconomic (SEC) status. The Index of Multiple Deprivation (IMD) is an area-based measure of relative deprivation, derived from the womens’ postcodes based on census statistics for overcrowded housing and other factors, and is believed to be the main confounding factor relating to both the exposure (i.e. the decision to attend screening), and the outcome.

#### ***Lead-time bias***

Lead-time is the amount of time by which the date of diagnosis has been advanced by screening detection as opposed to symptomatic occurrence. This confers a bias *in favour of screening*, which will be addressed at the analysis stage of case study 2 using two approaches: (1) “postponement of screen-detected cases” where the date of diagnosis for the screen-detected cases will be shifted forward for a period corresponding to the estimated lead-time (Gabe et al., 2007 [18]), and (2) statistical adjustment where the cases’ (and diseased controls, where applicable) additional follow-up time (between date of diagnosis and date of death/censor date) - observed purely as a result of lead-time - will be estimated by assuming an exponential distribution of the sojourn time, the period during which the tumour is asymptomatic but screen-detectable (Duffy et al., 2008b [19]). The estimates for mean sojourn time will be taken from Tabar et al. (2000 [20]).

Applying a restriction on the date of diagnosis during the selection of dead cases can also introduce a form of lead-time bias. Among cases who died from breast cancer on the same date, the screen-detected ones would have received their diagnosis earlier than the symptomatically-diagnosed cases, and therefore would tend to be excluded during the selection, introducing a bias in *favour of screening*. No restriction on the case’s date of diagnosis will be applied at the design stage, i.e. all women diagnosed after the inception of the NHSBSP, i.e. since 1990, will be included in the study.

#### ***Length bias***

Length-biased sampling occurs when the chance of an observation being in a sample is proportional to a particular characteristic of the observation. In the context of screening, length bias is the phenomenon whereby slower-growing, less aggressive tumours (including DCIS) have a longer pre-clinical screen-detectable period (PCDP) and are therefore more likely to be screen-detected compared to faster-growing, more aggressive cancers. This will confer a bias *in favour of screening*, which will be addressed at the analysis stage of case-study 2 by performing sensitivity analyses. Two latent tumour populations will be assumed, one – the “length bias group” - with both a higher probability of being screen-detected and a correspondingly lower probability of fatality, whether actually detected by screening or symptomatic (Duffy et al., 2008b [19]).

The extreme form of length bias is overdiagnosis, defined as the diagnosis of cancers which would not have come to clinical attention in the patient’s lifetime had screening not taken place. Overdiagnosis can be estimated by comparing cumulative incidence in screened and unscreened populations several years after screening stops (Biesheuvel et al., 2007 [21]). Estimates of overdiagnosis obtained from observational studies vary widely according to the design used, i.e. dynamic populations or cohort populations (Puliti et al., 2011 [16]). In case–control studies, which are nested within a cohort population, overdiagnosis can be robustly estimated provided that (1) the difference in underlying risk of primary breast cancer between screened and unscreened populations (i.e. self-selection bias), and (2) the period of time by which the diagnosis is brought forward by screening (i.e. lead-time bias) are both accounted for (Duffy et al., 2010 [13], Puliti et al, 2011 [16]). In the absence of overdiagnosis, the initial increase in primary breast cancer occurrence in the screened group would be fully compensated by a similar decrease in cancers among older age groups no longer offered screening (“compensatory drop method”, Puliti et al., 2011 [16]).

In addition to investigating the extent of the different biases when examining the relationship between screening and mortality or incidence, identifying homogeneous strata that may (positively or negatively) modify the effect of the screening exposure on the outcome (effect modifiers), such as age at diagnosis / pseudo-diagnosis (5-year band), epoch of diagnosis (5-year band), and tumour detection mode of the cases will be of interest.

### Statistical methods

All case–control study analyses will be conducted using conditional logistic regression. Matching factors (i.e. age, area) are controlled for in the design. All statistical analyses will be performed using the statistical software STATA or R.

**CC1.** The *primary objective* of this case–control will be to assess the effect of various measures of attendance at breast screening *strictly prior to* the case’s date of first diagnosis/ the control’s date of pseudo-diagnosis on mortality from primary breast cancer. The primary measure of attendance to screening will be whether a woman ever attended at least one screen episode (indicator variable). Secondary measures will be:

• the total number of screens (counts);

• the time since last screen (continuous variable), and whether that time span fell within the past three years (≤ 3 years or > 3 years), as this corresponds to the NHSBSP protocol and approximates the estimated PCDP;

• the time since penultimate screen (continuous or categorical);

• the interval between last screen and penultimate screen (continuous or categorical);

• the maximum interval between 2 screens (continuous or categorical);

• the average interval between 2 screens (continuous or categorical);

• the patient’s age at first screen (continuous or categorical);

• the patient’s age at last screen.

*Ascertainment, self-selection* and *exposure opportunity* biases will be addressed using the methods described in the *Bias & Effect modification* section.

***Secondary objectives*** will consider time since last screen stratified by age at diagnosis/pseudo-diagnosis, by first tumour detection mode, and by region (Main phase only).

The effect of attending a screen in a particular 3-year age band in the main phase (such as 47–49 inclusive), or 5-year age band in the pilot phase, on mortality from primary breast cancer in the subsequent 5-year age band (such as 50–54) will also be investigated, as performed for the cervical screening audit (Sasieni et al., 2009 [22]). The results of this analysis should be very similar to those obtained from the analysis of “time since last screen” after stratifying the analysis by age at diagnosis/pseudo-diagnosis.

**CC2.** The *primary objective* of this case–control will be to estimate the proportional effects of screening attendance *strictly prior to* date of diagnosis/pseudo-diagnosis and cancer treatment, on prognosis in patients diagnosed with primary breast cancer. The primary and secondary measures of attendance to screening will be as for CC1; in addition, they will include tumour detection mode to assess the relationship between detection mode and choice of cancer treatment.

The regression analysis will be adjusted for tumour attributes such as stage of disease at diagnosis and other pathological features. Because of the extreme collinearity between cancer treatment and pathology variables, e.g. lymph node positivity, a major determinant of treatment choice, estimating relative effects from the regression analysis often gives unreliable/biased estimates of cancer treatment (Wishart et al., 2010 [23]). To address this statistical issue, a counterfactual analysis will be performed (Höfler et al., 2005 [24]). The treatment that an individual actually does not receive is called counterfactual treatment, and the outcome under this treatment, after treatment assignment, is referred to as counterfactual outcome. In our study, the cancer treatment effects will be constrained to the absolute effect estimated from meta-analyses of the RCTs.

*Self-selection*, *lead-time, and length* biases will be addressed using the methods described in the *Bias & Effect modification* section. Because in CC2, both cases and controls have had primary breast cancer, *exposure opportunity* bias can be assumed to be minimal.

***Secondary objectives*** will consider time since last screen stratified by epoch of diagnosis to further explore collinearity among the explanatory variables.

**CC3a/b.** The *primary objectives* of these case-controls will be to estimate the effect attendance at breast screening strictly prior to date of diagnosis/pseudo-diagnosis on incidence of invasive (CC3a) or, of in situ and invasive (CC3b) primary breast cancer, and to estimate rates of overdiagnosis due to screening.

The primary and secondary measures of attendance to screening will be as for CC1, in particular time since last screen; in addition, they will include the patient’s age at last screen, and whether a woman ever missed attendance at recall assessment(s). This last measure should help assess potential room for improvement in the management of suspicious screening results.

With time since last screen, one might expect an observed increase in incidence (excess risk) immediately after a screen (the screen-detected cases), followed by an observed decrease in incidence (deficit in risk) due to the removal of pre-clinical cases by the screen. The deficit would be expected to be attenuated with time since the screen. The analysis will be stratified by age at diagnosis/pseudo-diagnosis. Indeed, one might expect an excess risk of disease in screened ages (within screening age range) and a deficit in risk in non-screened ages (above screening age range); a potential deficit in risk may also be seen at later ages within the screening age range (i.e. at age 65–69 after up to 15 years of screening). In the absence of overdiagnosis, the initial excess risk from screening would be fully compensated by a deficit in risk over time in the absence of screening (“compensatory drop”, Puliti et al., 2011 [16]). The excess risk in screened ages and the deficit in risk in non-screened ages will be quantified and combined with National Incidence Rates to construct risk scores (Field et al., 2005 [25]) in an attempt to estimate lifetime effect of screening on incidence and overdiagnosis. As the NHSBSP has been running for at least 20 years, there should be sufficient follow-up data after screening stopped (age 70), and the method of “compensatory drop” should achieve full adjustment for lead-time (Puliti et al., 2011 [16]). If the pilot phase of the study highlights little or no follow-up after last screen, lead-time bias will be adjusted using the “postponement of screen-detected cases” method (described in the *Bias & Effect modification* section).

The estimations will be performed for all disease (in situ and invasive) and for invasive disease only.

*Self-selection* and *exposure opportunity* biases will be also addressed using the methods described in the *Bias & Effect modification* section.

***Secondary objectives*** will consider time since last screen stratified by region (Main phase only).

**CC4.** The *primary objective* of this case–control will be to estimate the effect of attendance to breast screening *strictly prior to* date of diagnosis/pseudo-diagnosis on incidence of advanced stage primary breast cancer. The primary and secondary measures of attendance to screening will be as for CC1; in addition, they will include age at last screen.

*Self-selection* and *exposure opportunity* biases will be addressed using the methods described in the *Bias & Effect modification* section.

***Secondary objectives*** will consider time since last screen stratified by age at diagnosis/pseudo-diagnosis, and by region (Main phase only). Analysis may be adjusted for stage at diagnosis.

Results from CC1 and CC3 analyses will be combined with published data on breast cancer mortality and incidence over time, respectively, to estimate projected mortality, projected incidence, and stage-specific incidence in the presence and absence of screening and derive prevented fraction as an impact measure to help interpretation and evaluation of the programme benefits harms (Independent UK Panel on Breast Cancer Screening, 2012 [26]).

#### ***Ethics***

The study protocol was reviewed and approved by the UK department of health. Ethical approval was obtained from the London Research Ethics Committee (REC) of the National Research Ethics Service (NRES) (reference number 12/LO/1041), and by the National Information Governance Board NIGB) Ethics and Confidentiality Committee (ECC) (Reference number ECC 6–05 (e)/2012). Both organisations are now merged into the Confidential Advisory Group (CAG).

The study has received from the National Institute for Health Research Clinical Research Network (NIHR CRN) support, and has been provisionally assigned to the National Cancer Research Network (NCRN) (UKCRN Study ID = 14978, http://public.ukcrn.org.uk/search).

## Discussion

This protocol aims to evaluate the policy of mammography screening as delivered in the current NHSBSP in terms of, benefits on mortality from and on incidence of invasive primary breast cancer, and, harms from overdiagnosis. In the post-RCT epoch, analytical observational studies are the design of choice. The attraction of the case–control evaluation strategy resides in that, (1) it directly relates the clinical endpoint to the screening history at an individual level, (2) being retrospective in design it requires no further follow-up and is therefore quick to perform, and finally, (3) it needs a relatively small number of cases and corresponding controls. In addition, the case–control design allows the assessment of what actually happened in the population during service screening, taking into account natural variation. It also has the flexibility to question aspects of the screening regime which were not possible to address using RCT data. The case–control design is potentially prone to a number of biases, in particular some that could confer a bias in favour of screening. However, with careful design and analysis, one can minimize the risk of biased results. The case–control design proved indeed to be a powerful tool in the evaluation of the NHS Cervical Screening Programme, informing policy on screening intervals and age ranges (Sasieni et al., 2009 [22]).

## Abbreviations

CAG: Confidential advisory group; CCG: Clinical commissioning group; CN: Cancer network; DCIS: Ductal carcinoma in situ; DCO: Death certificate only; ECC: Ethics and confidentiality committee; HSCIC: Health & social care information centre; IMD: Index of multiple deprivation; KIT London: Knowledge & intelligence team London (formerly Thames Cancer Registry); LCIS: Lobular carcinoma in situ; NCDR: National cancer data repository; NCRN: National cancer research network; NHAIS: National health applications and infrastructure services; NIGB: National information governance board; NIHR CRN: National institute for health research clinical research network; NHSBCSP: National health service bowel cancer screening programme; NHSBSP: National health service breast screening programme; NHSCSP: National health service cervical screening programme; NRES: National research ethics service; ONS: Office for national statistics; PCDP: Pre-clinical screen-detectable period; PCT: Primary care trust; PRU: UK policy research unit in cancer awareness, screening and early diagnosis; RCTs: Randomised controlled trials; REC: Research ethics committee; SEC status: Socioeconomic status; SHA: Strategic health authority.

## Competing interests

The authors declare they have no competing interests.

## Authors’ contributions

NJM was responsible for the strategic aspects of design and statistical analyses of the study protocol, prepared the application to the NRES REC and NIGB ECC, led the submission, and wrote the manuscript. PDS provided methodological and practical expertise to the protocol from his experience with the NHS cervical screening programme. DP was responsible for the data management aspects of the study protocol and supported the submission to application to the NRES REC and NIGB ECC. SWD provided overall guidance to the study protocol design and statistical analyses. All authors read and approved the final manuscript.

## Pre-publication history

The pre-publication history for this paper can be accessed here:

http://www.biomedcentral.com/1471-2407/13/596/prepub
